# Supplementary material for: Adaptive federated clustering for uncertainty-aware learning on decentralized big data platforms
Source: PLoS One. 2025 Dec 1;20(12):e0337069. doi: 10.1371/journal.pone.0337069 (PMC12668549; doi:10.1371/journal.pone.0337069)
Supplement: S1 File — (DOCX) [file pone.0337069.s001.docx]

**APPENDIX:**

**(S1) Reproducibility Checklist:**

**Dataset Description:**

**Datasets Used:** **CIFAR-10, FEMNIST, IoT-Lab**

**Data Availability:**

- CIFAR-10: <https://www.cs.toronto.edu/~kriz/cifar.html>
- FEMNIST: <https://leaf.cmu.edu/>
- IoT-Lab: [Link to IoT-Lab Dataset](https://www.iot-lab.info/)

**Preprocessing Steps:** Data normalization, augmentation (rotation, flipping), and scaling to **[0,1]**.

**Experiment Settings:**

- Learning Rate: **0.01**
- Optimizer: **Adam**
- Batch Size: **32**
- Number of Rounds: **500** for CIFAR-10, **300** for FEMNIST, and **250** for IoT-Lab.
- Learning Rate Decay: **0.99** per 50 rounds.
- Loss Function: **Cross-Entropy Loss**.
- Gradient Clipping: **1.0** to prevent exploding gradients.

**Model Architecture Details:**

- **Encoder:** 4 Convolutional layers with Batch Normalization and ReLU activations.
- **Aggregator:** Hierarchical aggregation with weighted averaging.
- **Decoder:** Fully connected layers with softmax activation for multi-class classification.

**Privacy Mechanisms:**

- **Differential Privacy (DP):** Laplacian noise with ε = 1.0.
- **Homomorphic Encryption (HE):** Applied during aggregation.

**Hardware Configuration:**

- CPU: **Intel Xeon Gold 6130 @ 2.10GHz**
- GPU: **NVIDIA Tesla V100 (16GB VRAM)**
- RAM: **64GB DDR4**
- OS: **Ubuntu 20.04 LTS**
- Framework: **PyTorch 1.13.1**, **TensorFlow 2.11**

**Recommended GitHub Repositories:**

**1: Federated Learning with Homomorphic Encryption**

- **Repository:** [viensea1106/Federated-Learning-meets-Homomorphic-Encryption](https://github.com/viensea1106/Federated-Learning-meets-Homomorphic-Encryption)
- **Description:** This project proposes a privacy-preserving federated learning algorithm for medical data using homomorphic encryption. The algorithm employs a secure multi-party computation protocol to protect the machine learning model from adversaries. It includes an implementation plan utilizing PyTorch and the OpenFHE library. [GitHub+2GitHub+2GitHub+2](https://github.com/viensea1106/Federated-Learning-meets-Homomorphic-Encryption?utm_source=chatgpt.com)

**2: Fed-DP: Federated Learning Platform**

- **Repository:** [NigeloYang/Fed-DP](https://github.com/NigeloYang/Fed-DP)
- **Description:** Fed-DP provides a foundational platform for beginners intending to start with federated learning. It integrates differential privacy mechanisms and offers modules for model optimization, data partitioning, and more. The framework is designed to be highly customizable and includes plans to incorporate homomorphic encryption. [GitHub](https://github.com/NigeloYang/Fed-DP?utm_source=chatgpt.com)

**3: Simulating Federated Learning with Homomorphic Encryption**

- **Repository:** [aayushsss1/Simulating-Federated-Learning-with-Homomorphic-Encryption](https://github.com/aayushsss1/Simulating-Federated-Learning-with-Homomorphic-Encryption)
- **Description:** This repository contains code and resources for simulating federated learning with homomorphic encryption using a Streamlit app and Flask. It demonstrates training machine learning models across clients while preserving data privacy through homomorphic encryption. [GitHub](https://github.com/aayushsss1/Simulating-Federated-Learning-with-Homomorphic-Encryption?utm_source=chatgpt.com)

**4: Differential Privacy-Based Federated Learning**

- **Repository:** [wenzhu23333/Differential-Privacy-Based-Federated-Learning](https://github.com/wenzhu23333/Differential-Privacy-Based-Federated-Learning)
- **Description:** This repository collects related papers and corresponding codes on differential privacy-based federated learning. It includes personal implementations and aims to support discussions and improvements in the field. [GitHub](https://github.com/wenzhu23333/Differential-Privacy-Based-Federated-Learning?utm_source=chatgpt.com)
