# Supplementary material for: Adaptive federated clustering for uncertainty-aware learning on decentralized big data platforms
Source: PLoS One. 2025 Dec 1;20(12):e0337069. doi: 10.1371/journal.pone.0337069 (PMC12668549; doi:10.1371/journal.pone.0337069)
Supplement: S1 Table — (DOCX) [file pone.0337069.s002.docx]

**(S2) Table-S2-1 Experimental Setup:**

| **Parameter** | **Value** |
| --- | --- |
| Dataset Used | CIFAR-10, FEMNIST, IoT-Lab |
| Model Architecture | 4-layer CNN for CIFAR-10, LSTM for FEMNIST, ResNet-18 for IoT-Lab |
| Number of Clients | 100 (10 selected per round) |
| Communication Rounds | 500 |
| Local Epochs | 5 |
| Learning Rate | 0.01 |
| Optimizer | Adam |
| Batch Size | 32 |
| Gradient Clipping | 1.0 |
| Differential Privacy | Laplacian Mechanism, ϵ=1.0\epsilon = 1.0ϵ=1.0 |
| Homomorphic Encryption | Paillier Cryptosystem |
| Backdoor Detection | L2 Norm Threshold δ=0.8\delta = 0.8δ=0.8, Cosine Similarity τ=0.75\tau = 0.75τ=0.75 |
| Hardware Configuration | Intel Xeon CPU, NVIDIA Tesla V100 GPU, 64 GB RAM |
| Framework | PyTorch 1.13.1, TensorFlow 2.11 |
| OS | Ubuntu 20.04 LTS |
