# Supplementary material for: Adaptive federated clustering for uncertainty-aware learning on decentralized big data platforms
Source: PLoS One. 2025 Dec 1;20(12):e0337069. doi: 10.1371/journal.pone.0337069 (PMC12668549; doi:10.1371/journal.pone.0337069)
Supplement: S2 Table — (DOCX) [file pone.0337069.s003.docx]

**(S3) Table-S3-1 Data Partitions:**

| **Dataset** | **Partitioning Method** | **Training Clients** | **Testing Clients** |
| --- | --- | --- | --- |
| CIFAR-10 | IID and Non-IID splits | 80 | 20 |
| FEMNIST | User-based partitioning (by writer) | 100 | 20 |
| IoT-Lab | Geographical split by IoT devices | 150 | 30 |

**Partitioning Details:**

**IID Partitioning:** Data samples are evenly distributed among clients, ensuring each client receives a balanced subset of classes.

**Non-IID Partitioning:** Some clients receive samples from a limited number of classes, mimicking real-world device skew.

**User-based Partitioning:** FEMNIST is partitioned by individual writers, creating natural non-IID distributions.

**Geographical Split:** IoT-Lab devices are grouped based on their physical locations to simulate regional deployments.

**Hyperparameters:**

| **Hyperparameter** | **Value** |
| --- | --- |
| Learning Rate | 0.01 |
| Batch Size | 32 |
| Local Epochs | 5 |
| Gradient Clipping | 1.0 |
| Privacy Budget (ε) | 1.0 |
| Noise Scale (DP) | 0.5 |
| Encryption Key Size | 2048 bits (Paillier Cryptosystem) |
| Backdoor Detection Threshold | 0.8 (L2 Norm), 0.75 (Cosine Similarity) |
| Communication Rounds | 500 |
