# Supplementary material for: Adaptive federated clustering for uncertainty-aware learning on decentralized big data platforms
Source: PLoS One. 2025 Dec 1;20(12):e0337069. doi: 10.1371/journal.pone.0337069 (PMC12668549; doi:10.1371/journal.pone.0337069)
Supplement: S2 File — (DOCX) [file pone.0337069.s004.docx]

**(S4) Long-Term Stability Analysis (500 Rounds)**

**Data for Long-Term Stability Analysis (500 Rounds)**

| **Training Round Interval** | **Accuracy (%)** | **Communication Overhead (MB)** | **Model Convergence (%)** |
| --- | --- | --- | --- |
| 0 - 50 | 81.5 | 4.8 | 25 |
| 50 - 100 | 83.2 | 9.3 | 50 |
| 100 - 200 | 85.4 | 18.5 | 75 |
| 200 - 300 | 86.8 | 27.1 | 87 |
| 300 - 400 | 87.6 | 34.9 | 94 |
| 400 - 500 | 88.3 | 42.5 | 98 |

The Long-Term Stability Analysis confirms the robustness of the proposed model over 500 training rounds. Accuracy steadily improves from 81.5% in the initial rounds to 88.3% by the 500th round, demonstrating consistent learning. The communication overhead grows predictably, reaching 42.5 MB at completion, indicating efficient bandwidth usage. The model achieves 98% stability, reflecting strong convergence and reliability across extended training cycles. These results validate the model's capacity for sustainable performance and efficient communication in federated learning scenarios.
