# Supplementary material for: Adaptive federated clustering for uncertainty-aware learning on decentralized big data platforms
Source: PLoS One. 2025 Dec 1;20(12):e0337069. doi: 10.1371/journal.pone.0337069 (PMC12668549; doi:10.1371/journal.pone.0337069)
Supplement: Attachment — Submitted filename: V2-Response File.docx [file pone.0337069.s006.docx]

## Rebuttal Report

### **Reviewer #1 Comments**

**Comment 1:**
However, it is advised to align the problem statement and the objectives.

**Response:**
I have revised Section **1.2 Problem Definition** and **Objectives** to ensure that each problem statement directly maps to a corresponding objective.

**Comment 2:**
It is strongly advised to add the latest references for each of the problem statements. For example, the problem statement one is referring to inadequate approaches for large-scale, decentralized big data. It would be more verifiable if comes with a few latest references.

**Response:**
Latest references from 2022–2024 have been added in the **Problem Definition** section to support each identified problem.

**Comment 3:**
Also make sure the objective one addressing the first problem statement. The objective two addressing the second problem statement and so on.

**Response:**
The objectives have been reordered and rewritten so that each directly addresses its corresponding problem statement in Section **1.2**.

### **Reviewer #2 Comments**

***Comment 1:***	*The abstract is written in a very general way and does not provide enough detail. It also lacks clear evidence and results to show the real importance of the work. Adding more technical explanation, presenting solid results, and mentioning possible limitations would make the abstract stronger.*

**Response:**
The **Abstract** has been revised to include technical details, quantitative results, and a brief note on limitations.

***Comment 2:***	*The paper covers an interesting topic but has many grammar and writing style problems. These issues affect readability. For example, in one sentence an extra number “53” appeared by mistake, and the phrase could be written more smoothly. The authors should carefully correct grammar and remove such errors to improve clarity.*

**Response:**
The manuscript has been carefully proofread. Grammar issues, typos, and stylistic errors (including the extra “53”) have been corrected throughout the document.

***Comment 3:***	*The paper discusses a relevant issue, but the problem statement and research objectives are not clearly explained. The contributions of the study should also be highlighted more directly. The introduction and overall structure need better organization to improve the flow of ideas. There are also several grammar and typo errors. With clearer writing and better structure, the paper has strong potential.*

**Response:**
I revised the **Introduction** for better organization, clarified the **Problem Definition**, aligned the **Objectives**, and explicitly highlighted the **Contributions**. Grammar and typographical errors were also corrected.

***Comment 4:***	*The experiments are incomplete because important benchmark datasets like CIFAR-10, CIFAR-100, and Fashion-MNIST are missing. These are standard datasets in the field and should be included to test the method properly. In addition, the results are not compared with existing state-of-the-art methods. Without these comparisons, it is hard to judge the true performance. The paper would be stronger if it included experiments with these datasets and provided comparisons with baseline methods.*

**Response:**
I expanded the **Experimental Section** by adding results on CIFAR-10, CIFAR-100, and Fashion-MNIST. Comparisons with FedAvg, FedProx, FedYogi, FedAdapt, FedFormer, and HAFED have also been included.

***Comment 5:***	*The paper uses the term “vague system boundaries” but does not explain it clearly. Readers do not know what these boundaries mean in the context of federated learning. The authors should explain whether it relates to differences between clients, the scope of data, or something else. A clear explanation would help readers understand the challenges and how AFC deals with them.*

**Response:**
I expanded Section **1.2 Problem Definition** and the related discussion to clarify vague system boundaries. The explanation now covers client roles, participation stability, and data ownership, with healthcare and IoT examples.

***Comment 6:***	*The paper introduces the AFC framework but the introduction section has many issues. The structure is unclear, and there is no roadmap to guide the reader. Subheadings appear suddenly and disrupt the flow, while too much technical detail is placed too early in the paper. The problem statement is scattered and unclear, and the AFC framework is introduced too late, making the contribution less visible.*

**Response:**
I reorganized the **Introduction**. A clear roadmap is now provided. Technical details were shifted to the **Methodology**, while the **Problem Statement** and **Objectives** are presented in a structured manner.

***Comment 7:***	*The motivation for AFC is also not strong enough, as the introduction does not show why AFC is needed compared to existing research. Transitions between sections are abrupt, and the objectives and contributions are not clearly linked to the challenges. The introduction should be reorganized so that the challenges flow smoothly into the problem statement, followed by the proposed AFC framework. The objectives should be listed clearly, and the novelty of AFC should be emphasized.*

**Response:**
The **Introduction** has been rewritten to strengthen motivation. Section flow was improved so that challenges lead directly into the problem statement, objectives, and then the AFC framework. The novelty of AFC is emphasized with explicit comparisons to prior work.

***Comment 8:***	*There are also minor issues such as grammar errors, awkward sentences, and too much technical detail in the introduction that should be moved to the related work section. The paper also does not discuss any limitations of AFC, which would help present a more balanced perspective. Overall, the paper has promise, but the introduction needs major revision to improve clarity, structure, and motivation.*

**Response:**
I corrected grammar and sentence structure issues, moved technical details from the **Introduction** to **Related Work**, and added a **Limitations and Future Work** section.

***Comment 9:***	*Figure 2 does not clearly show the concept of adaptive federated clustering and selection. The labeling is confusing and the arrows are repetitive. The explanation in the methodology section also does not clarify the figure well. Both the figure and the related text should be revised for better understanding.*

**Response:**
**Figure 2** has been redesigned with clearer labels and simplified arrows. The explanation in the **Methodology Section** was rewritten to align directly with the figure.

**Comment 10:**
The dataset section is weak because it does not explain why the chosen dataset, MIMIC-III, was selected. The connection between the dataset and the main concept of the paper is not clear. The authors should explain how the dataset relates to the study and why it is suitable.

**Response:**
I expanded the **Dataset Section** to justify the selection of MIMIC-III. The description now explains its relevance to healthcare, decentralized silos, and privacy-preserving FL scenarios.

## **Summary:**

I sincerely thank the reviewers for their detailed and constructive feedback. All comments were carefully addressed through major revisions, including structural improvements, grammar corrections, expanded experimental evaluation, clarification of vague concepts, and stronger motivation. I believe the revised manuscript is now significantly improved in clarity, technical depth, and alignment with the journal’s standards.
